# Early outcomes of augmented glenoid components in anatomic total shoulder arthroplasty: a systematic review

**DOI:** 10.1177/17585732211032922

**Published:** 2021-08-18

**Authors:** Ujash Sheth, James YJ Lee, Diane Nam, Patrick Henry

**Affiliations:** Sunnybrook Orthopaedic Upper Limb, Sunnybrook Health Sciences Centre, Division of Orthopaedic Surgery, University of Toronto, Toronto, Ontario, Canada

**Keywords:** Augmented glenoid, anatomic total shoulder arthroplasty, posterior glenoid deficiency

## Abstract

**Background:**

The objective was to evaluate the short-term clinical and radiological outcomes following augmented anatomic total shoulder arthroplasty in patients with posterior glenoid deficiency.

**Methods:**

An electronic search of EMBASE, MEDLINE, and PubMed identified studies reporting clinical and radiographic outcomes following augmented anatomic total shoulder arthroplasty among patients with posterior glenoid deficiency.

**Results:**

Nine studies including 312 shoulders underwent anatomic total shoulder arthroplasty using an augmented glenoid implant between 2015 and 2020. A statistically significant improvement in range of motion (ROM), visual analog scale (VAS), American Shoulder & Elbow Surgeons (ASES), Constant, University of California - Los Angeles and Simple Shoulder Test (SST) scores was demonstrated at mean follow-up of 37.1 months. Glenoid retroversion improved from 21.8° to 9.5°. At final follow-up, radiolucency was reported in 35.1% of shoulders. The 16° full-wedge augment led to higher and more severe radiographic lucency, while high peg perforation rates (44%) were observed among 5-mm augment stepped implants. The overall rate of complication was 2.6%. Rate of revision surgery was 1.9%.

**Conclusions:**

Overall, early- to mid-term outcomes following augmented anatomic total shoulder arthroplasty for posterior glenoid deficiency demonstrate good to excellent overall clinical results. More radiographic and clinical failures were reported in larger full wedge (16°) augments and stepped augments (5 mm). Prospective studies examining mid- and long-term outcomes will help further elucidate safety and efficacy of these relatively new implants.

## Introduction

Shoulder arthroplasty is the third most frequently performed joint replacement procedure in the United States.^1,2^ The overall incidence of shoulder arthroplasty is increasing at a greater rate than total hip and knee arthroplasty with predictive models estimating over 174,000 procedures performed annually by the year 2025.^3^ Anatomic total shoulder arthroplasty (aTSA) is indicated in primary glenohumeral joint osteoarthritis, inflammatory arthritis and post-traumatic arthritis.^4^ Management of the glenoid in primary aTSA can be challenging due to eccentric bone loss and increased retroversion, which have been associated with early glenoid loosening.^5,6^ In fact, aseptic loosening of the glenoid component is the most common mode of failure in aTSA.^7^

The Walch Classification is based on the mid-glenoid axial slice of a computed tomographic scan and is used to describe glenoid morphology in commonly occurring wear patterns.^8,9^ Posterior subluxation of the humeral head is the hallmark feature of a Type B glenoid, which is classified into three subgroups. Type B2 has posterior glenoid erosion resulting in a biconcave glenoid comprised of a neoglenoid and paleoglenoid, and type B3, which was an addition to the original classification, has continued preferential posterior wear leading to a monoconcave glenoid with retroversion greater than 15° and/or humeral head subluxation of greater than 70%.

Various techniques have been utilized to address the excessive retroversion caused by posterior erosion in type B2 and B3 glenoid morphology. These include hemiarthroplasty, eccentric reaming of the high side, posterior glenoid bone grafting, the use of reverse total shoulder arthroplasty (rTSA), and posteriorly augmented glenoid components. Hemiarthroplasty fails to correct posterior glenohumeral subluxation, resulting in persistent pain and further glenoid wear, ultimately requiring early conversion to a total shoulder prosthesis.^10–12^ Eccentric reaming is limited by the amount of retroversion correction achieved, as a correction of greater than 15° has been associated with penetration of the glenoid vault secondary to compromised anterior subchondral bone stock.^13,14^ Posterior glenoid bone grafting is technically demanding and carries the risk of nonunion, resorption, and subsidence. In fact, a recent study demonstrated a graft failure rate of 17% and a revision rate of 14% at a mean follow-up of over five years.^15–18^ Finally, the constrained nature of a rTSA helps address the posterior instability associated with the B2 and B3 glenoid.^19^ However, its use does not preclude the need for eccentric reaming or bone grafting. Moreover, there remains a paucity of long-term data on the outcomes following rTSA in patients with B2 and B3 glenoids, particularly among young, active patients, who are frequently the ones presenting with these wear patterns.^20–22^

**Figure 1.**
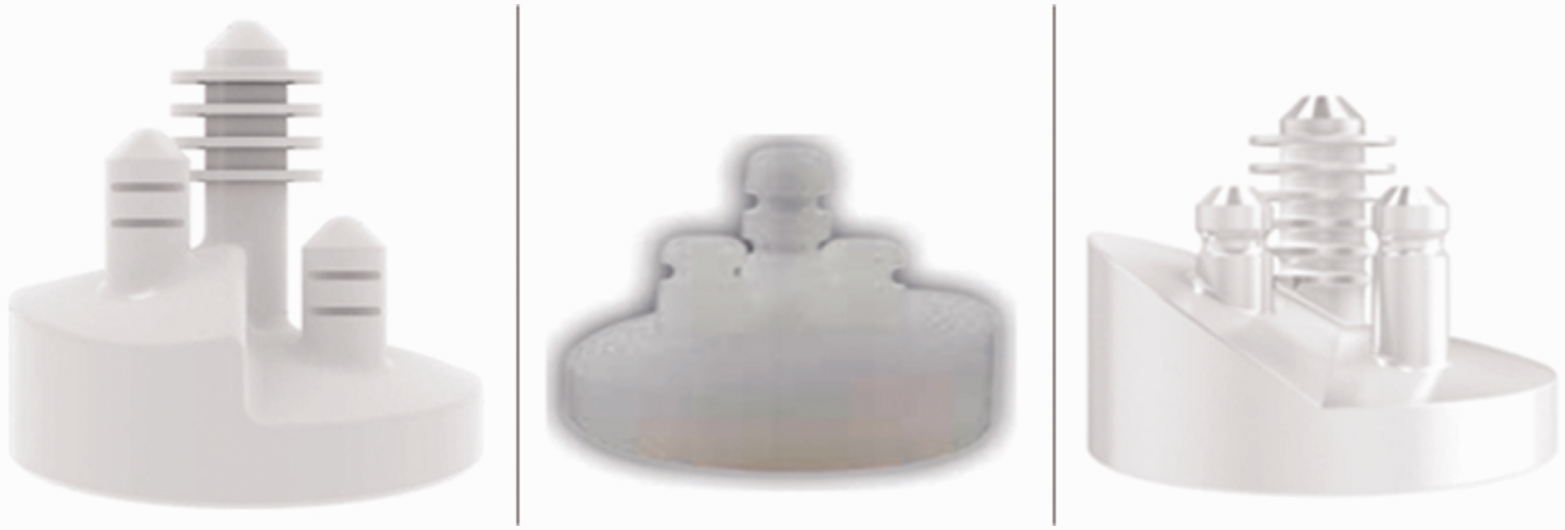
Augmented glenoid implants. Left: Stepped implant (Global StepTech; DePuy Synthes, Warsaw, IN, USA), Middle: Full-wedge glenoid (Equinoxe; Exactech, Gainesville, FL, USA). Right: Half-wedge glenoid (Aequalis Perform+; Wright Medical Group, Memphis, TN, USA).

Augmented glenoid components allow for retroversion correction while limiting excessive reaming, preserving bone stock and avoiding joint medialization, thus, preserving length–tension relationships and optimizing muscle function and joint stability.^23^ They also provide the theoretical benefit of improving implant longevity by lowering the risk of early loosening by decreasing edge loading, eccentric loading, and shear and tensile stresses at the bone–implant, cement–implant, and cement–bone interfaces.^23–26^ Currently available augmented glenoid components have an all-polyethylene monoblock design featuring a full-wedge, half-wedge, or step built into the backside of the component (Figure 1; modified from work by Friedman et al.).^23^ The full-wedge glenoid component features a complete wedge from anterior to posterior and allows for 8°, 12°, or 16° of retroversion correction (Equinoxe; Exactech, Gainesville, FL, USA).^27^ The half-wedge glenoid has 15°, 25°, and 35° augments on the posterior half of the implant which correct retroversion by 7°, 12°, and 17°, respectively (Aequalis Perform+; Wright Medical Group, Memphis, TN, USA).^27^ Stepped implants have a stepped surface that contacts the prepared native bone surface perpendicular to the joint and are available in three sizes: +3 mm, +5 mm, and +7 mm, which correspond to 10°, 15° and 20° corrections, respectively (Global StepTech; DePuy Synthes, Warsaw, IN, USA).^27^ While finite element analyses and biomechanical studies support the use of posterior augmented glenoid components, there is a paucity of literature on the clinical outcomes following augmented aTSA. As a result, the objective of this review was to present the short-term clinical and radiological outcomes following augmented aTSA in patients with posterior glenoid wear patterns.

## Materials and methods

This systematic review of the peer-reviewed literature was conducted according to the Preferred Reporting Items for Systematic Reviews (PRISMA) guidelines.^28^ All studies reporting on clinical and radiological outcomes following augmented aTSA in patients with posterior glenoid wear patterns were eligible for inclusion. An electronic search using EMBASE, MEDLINE and PubMed was conducted through 4 February 2020 using the following MeSH search terms: “augmented glenoid,” “shoulder arthroplasty,” “arthroplasty,” “replacement,” “shoulder,” and “osteoarthritis.” Additional studies were identified by reviewing the reference lists of eligible articles.

Studies were excluded if they reported on discontinued implants, metal augments, rTSA, biomechanical studies, and revision procedures. Additional exclusion criteria included a mean follow-up of less than two years for clinical outcomes. A shorter duration of follow-up was accepted for evaluation of radiographic parameters (i.e., retroversion correction). There were no language restrictions.

**Figure 2.**
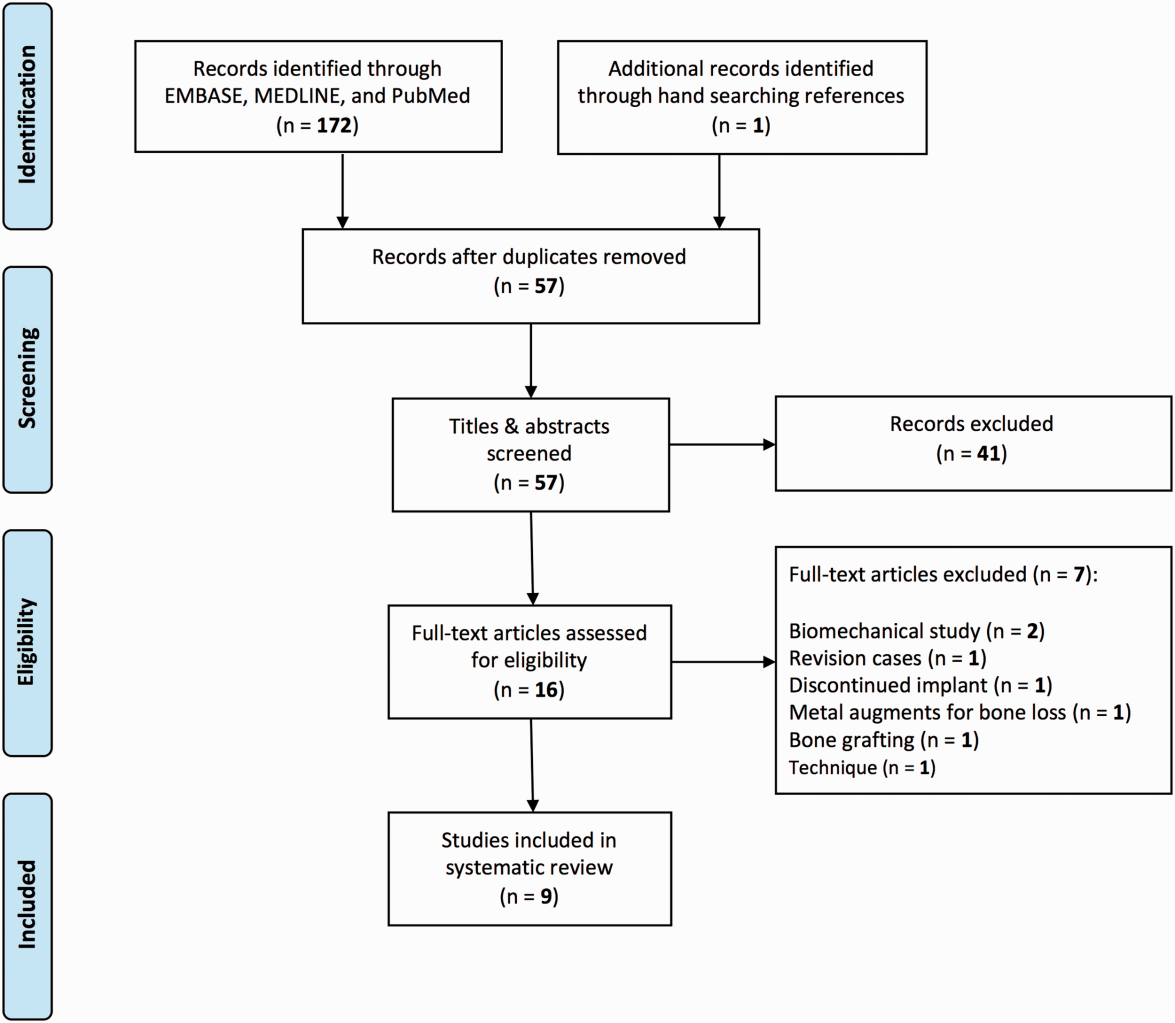
PRISMA flow diagram.

A total of 173 articles were identified from the initial electronic search, ultimately, nine articles were eligible for inclusion (Figure 2). Data were extracted independently by two reviewers. Data collected from studies included: author, year of publication, sample size, sex and age of participants, implant used, duration of follow-up, patient-reported outcome measure (PROM) used, and radiographic parameters.

## Results

### Demographic characteristics

A total of 312 shoulders in 308 patients underwent aTSA using an augmented glenoid component between 2015 and 2020. Three studies directly compared outcomes following aTSA with augmented (N = 110) and non-augmented glenoid components (N = 109).^29–31^ The six remaining studies were case series’. The mean age of patients was 65.1 years (range, 37–81 years), with males comprising 68% of the sample. The mean duration of follow-up was 37.1 months (range, 2.3–72 months). Three studies utilized a full wedge (Equinoxe; Exactech, Gainesville, FL, USA), four studies used a stepped implant (Global StepTech; DePuy Synthes, Warsaw, IN, USA), and two studies used a half wedge component (Aequalis Perform+; Wright Medical Group, Memphis, TN, USA).^29–37^ Study characteristics of included studies are summarized in Table 1.

**Table 1.** Summary of included studies on augmented glenoid use in anatomic total shoulder arthroplasty.

Author (year)	Level of evidence	Sample size		Sex (M:F)	Mean age, years (SD)		Mean follow-up, months (SD)	Glenoid type (Walch)		Mean Preoperative glenoid retroversion (°)	Implant design (manufacturer)	Outcome measures
		Augmented glenoid (N)	Non- Augmented glenoid (N)		Augmented glenoid	Non- Augmented glenoid		Augmented glenoid (N)	Non- augmented glenoid (N)			
Thomas and Wright (2015)	III	24	24	34:14	65.8 (11.5)	66.4 (9.1)	29.5 (8.2)	NR	NR	NR	Full-Wedge Equinoxe (Exactech)	SST UCLA ASES CMS SPADI ROM (FE/AB/ER/IR) SASI
Favorito et al. (2016)	IV	22 (*19 patients*)	–	15:4	62.0 (8.3)	–	36.0 (5.0)	B2 = 20 C = 2	–	23.5	Stepped Global StepTech (DePuy)	WOOS VAS SF-36 ROM (FE/ER) Radiolucency Component seating Central peg integration
Stephens et al. (2017)	IV	21	–	NR	66.0 (5.8)	–	35.0 (4.3)	B2 = 19 C = 2	–	20.8	Stepped Global StepTech (DePuy)	SST ASES VAS ROM (FE/ER) Radiolucency component seating Glenoid version Correction Central peg integration
Ho et al. (2018)	IV	71	–	55:16	65.0 (7.0)	–	28.8 (11.4)	B2 = 46 B3 = 25	–	24.0	Stepped Global StepTech (DePuy)	PSS VR-12 ROM (FE/ER) Glenoid Version Correction Central Peg Integration
Ko et al. (2019)	III	49	48	61:36	67.4 (NR)	65.3 (NR)	48 (NR)	B1 = 3 B2 = 37 C = 9	B1 = 8 B2 = 33 C = 7	27.1	Stepped Global StepTech (DePuy)	Glenoid Version Correction Central Peg Perforation
Priddy et al. (2019)	III	37	37	52:22	64.1 (10.3)	65.5 (8.8)	38.4 (12.0)	A1 = 1 A2 = 2 B1 = 1 B2 = 27 B3 = 5 C = 1	A1 = 7 A2 = 8 B1 = 4 B2 = 11 B3 = 7 C = 0	NR	Full-Wedge Equinoxe (Exactech)	UCLA ASES CMS VAS ROM (FE/AB/ER/IR) Radiolucency
Das et al. (2020)	IV	11 (*10 patients*)	–	5:5	59 (10.2)	–	4.8 (4.9)	B2 = 11	–	16.0	Half-Wedge Aequalis Perform + (Tornier)	Glenoid Version Correction Humeral Scapular Offset
Grey et al. (2020)	IV	68	–	44:24	64.9 (7.2)	–	49.9 (18.2)	B1 = 10 B2 = 46 B3 = 12	–	17.3	Full-Wedge Equinoxe (Exactech)	SST UCLA ASES CMS SPADI ROM (FE/AB/ER/IR) Radiolucency Glenoid Version Correction
Terrier et al. (2020)	IV	9	–	4:5	68.8 (8.9)	–	3.5 (2.6)	B2 = 5 B3 = 4	–	17.3	Half-Wedge Aequalis Perform + (Tornier)	Glenoid Version Correction Scapulohumeral Subluxation

SD: standard deviation; SST: Simple Shoulder Test; UCLA: University of California (Los Angeles); ASES: American Shoulder & Elbow Society; CMS: Constant-Murley Score; SPADI: Shoulder Pain and Disability Index; ROM: range of motion; FE: forward elevation; AB: abduction; ER: external rotation; IR: internal rotation; SASI: Shoulder Arthroplasty Subluxation Index; WOOS: Western Ontario Osteoarthritis of the Shoulder score; VAS: visual analog scale; SF-36: short-form 36; PSS: Penn Shoulder Score; VR-12: Veterans-RAND 12; NR: not reported.

### Patient-reported outcome measures

**Figure 3.**
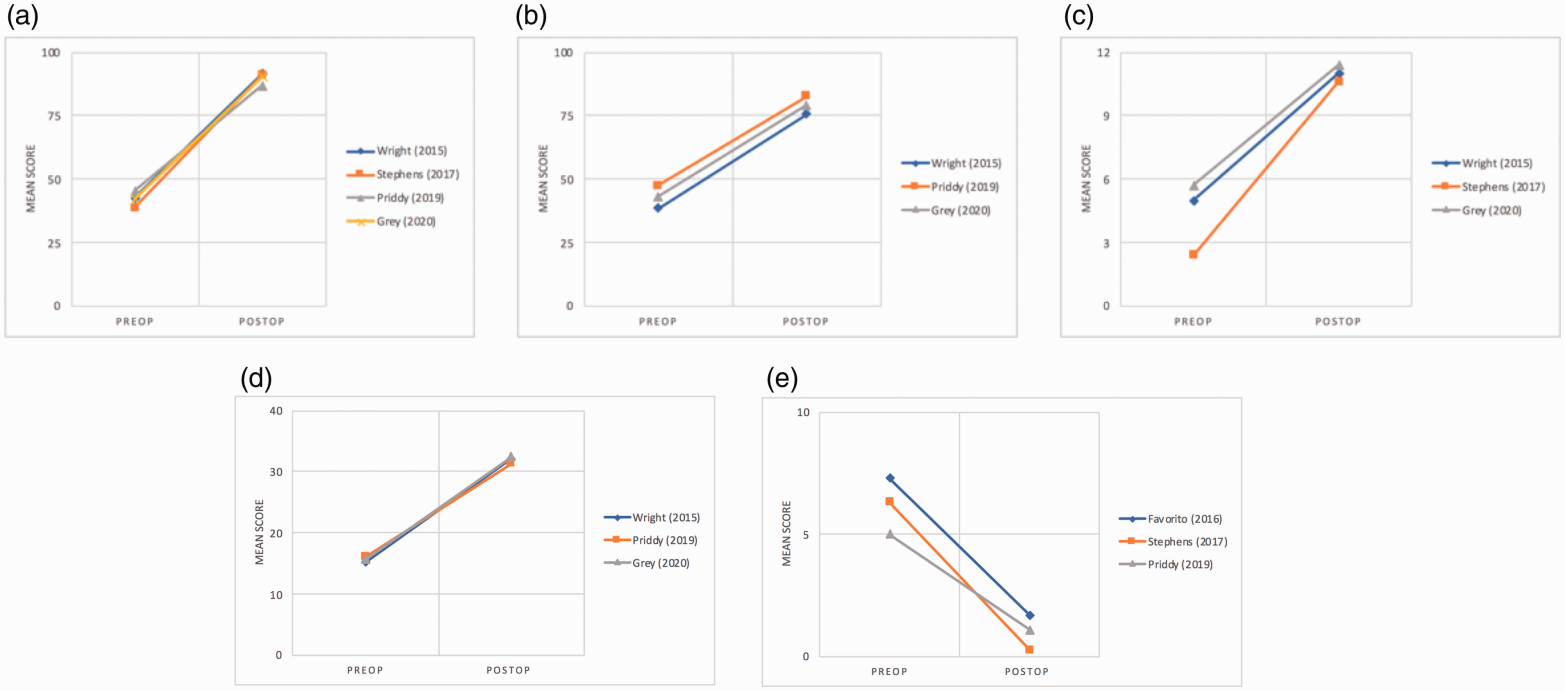
Slopegraph of patient-reported outcome measures. (a) ASES. (b) Constant score. (c) SST. (d) UCLA. (e) VAS. ASES: American Shoulder & Elbow Society; SST: Simple Shoulder Test; UCLA: University of California (Los Angeles); VAS: visual analog scale.

There was considerable heterogeneity among PROMs utilized between the nine eligible studies. The American Shoulder Elbow Society (ASES) score was the most frequently used PROM. Four studies^29,31,33,36^ with a combined sample size of 68 shoulders included the ASES score, which uniformly demonstrated an improvement in pre- to post-operative scores. Similar improvements were observed in the Constant, University of California (Los Angeles) (UCLA), and simple shoulder test (SST) scores (Figure 3). The mean Visual Analog Scale (VAS) score from three studies^31–33^ improved from 5.9 pre-operatively to 1.1 post-operatively. Two studies^29,36^ utilized the shoulder pain and disability index (SPADI) and observed a decrease in the mean score from 77 pre-operatively to 11.2 following augmented aTSA. Due to the lack of consistent PROMs used among the include studies, results could not be stratified by augmented component design (i.e., full wedge, half wedge, or stepped).

### Range of motion

**Figure 4.**
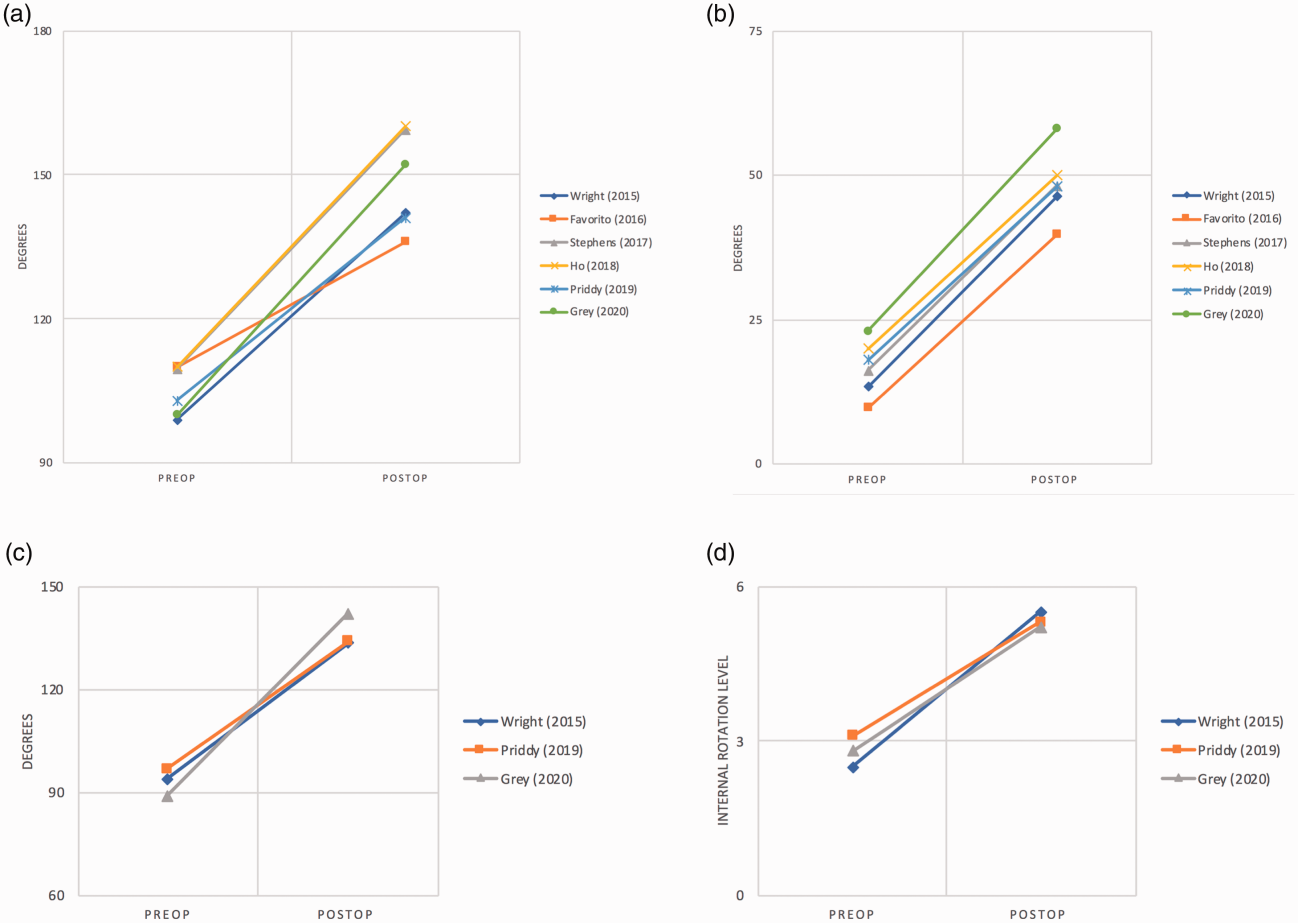
Slopegraph of range of motion. (a) Forward elevation. (b) External rotation. (c) Abduction. (d) Internal rotation.

Six studies including 243 shoulders examined pre-operative and post-operative forward elevation and external rotation, while three studies (129 shoulders) recorded abduction and internal rotation. Mean pre-operative forward elevation was 105°, which improved 45.9° to 150.9° post-operatively. Abduction increased from 92.2° to 138.1°. External rotation rose an average of 31.9°, from 18.6° to 50.5° while internal rotation showed an improvement from a mean score of 2.8 to 5.3 post-operatively ([1] Trochanter, [2] Buttock, [3] Sacrum, [4] L5-L4, [5] L3-L1, [6] T8-T12, [7] T7, or higher) (Figure 4).

### Radiographic outcomes

**Figure 5.**
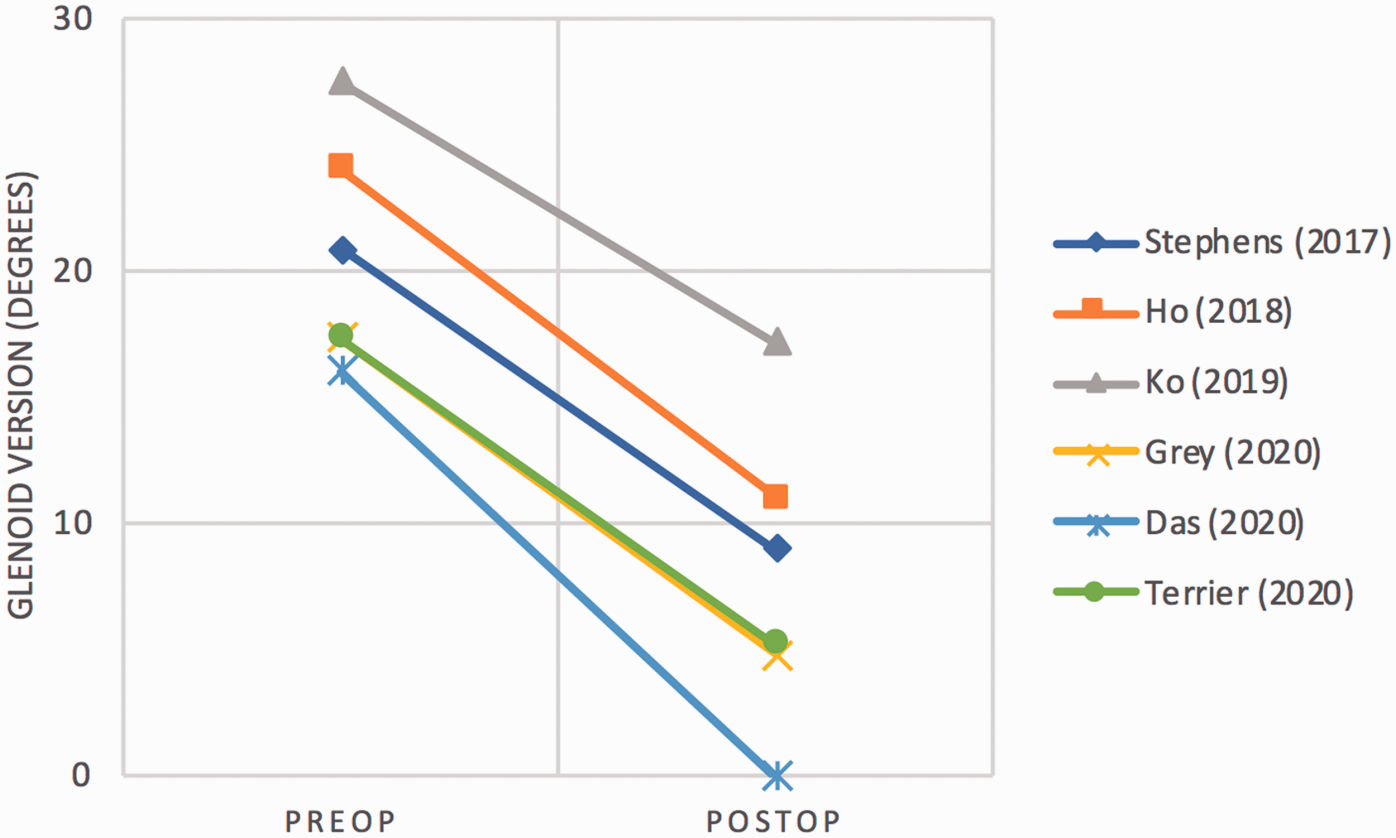
Slopegraph of glenoid retroversion correction.

The mean pre-operative glenoid retroversion reported in six studies^30,33–37^ was 21.8°, with an average correction of 11.3° to 9.5° of retroversion post-operatively (Figure 5). Radiolucency around the glenoid component was noted in 37% of shoulders (four studies,^31–33,36^ 148 patients); however, two-thirds of these cases had a Lazarus radiolucency grading^38^ of I and II (i.e., incomplete radiolucency and complete radiolucency around one peg only). Center peg radiolucency was described in four studies, all of which utilized a stepped glenoid component (Global StepTech; DePuy Synthes, Warsaw, IN, USA). A total of 19 peg perforations (11.7%) were noted. Among the 113 shoulders in which extent of osteointegration was graded, 14 shoulders (12.4%) demonstrated osteolysis around the peg (grade I), while the remaining 98 shoulders (87%) were found to have grade II (bone growth to edge of flanges) or III (bone growth within flanges) changes at mean follow-up of 36.3 months.

### Complications

A total of eight complications were reported in the eligible studies.^29–34,36^ Favorito et al.^32^ noted an anterior dislocation in a patient two weeks post-operatively, requiring revision to a larger head. One patient suffered two posterior dislocations, 22 months and 30 months following surgery requiring revision to a rTSA. Priddy et al. reported a prosthetic joint infection two and a half years post-operatively and a case of aseptic glenoid loosening three years post-operatively, both required revision procedures. In both cases, the 16° augment was utilized. Grey et al.^36^ noted two cases of glenoid component loosening in Walch B2 glenoids requiring revision and two cases of axillary neuropraxia. A radiographic study by Ko et al.^30^ reported a high peg perforation rate (44%) for the 5-mm stepped augment design (Global StepTech; DePuy Synthes, Warsaw, IN, USA). The authors did not find a statistically significant association between peg perforation and pre-operative retroversion or the amount of version correction.

## Discussion

Classically, aTSA in the setting of a severely posteriorly deficient glenoid has been a challenging clinical scenario for shoulder arthroplasty surgeons. In shoulders with a high degree of retroversion, it is widely accepted that anterior glenoid reaming beyond 15° poorly corrects posterior subluxation and is associated with a high risk of peg perforation and anterior rim fracture.^39^ Current commercially available posterior augmented glenoid implants aim to provide glenoid version correction while minimizing vault perforation, joint line medialization and excessive subchondral bone removal. The present systematic review identified nine recent studies comprising of 312 shoulders that underwent aTSA with an augmented glenoid component to address posterior bone deficiency, with 21.8° of mean pre-operative retroversion. Although the heterogeneity in outcome measures limited our ability to directly compare the three currently available implant designs (i.e., full wedge, half wedge, and stepped components), the short-term outcomes of these augmented components have been promising, with no evidence of superiority of one design over another.

Radiographic complications following aTSA has been a topic of interest within the shoulder arthroplasty literature. Walch et al.^6^ retrospectively analyzed 85 biconcave glenoids treated with aTSA and eccentric reaming at a mean follow-up of 77 months and found high rates of complication. They noted definite radiographic loosening in 19 shoulders (20.6%) with 15 revisions (16.3%) performed for aseptic glenoid loosening (6.5%), posterior instability (5.5%), or soft tissue problems (4.3%). A statistically significant correlation between radiographic loosening and increasing glenoid retroversion, posterior wear and subluxation was observed in this study. Walch and colleagues also found that neoglenoid retroversion had the strongest predictive value for postoperative complications. However, a recent study by Grantham et al.^40^ has challenged this view. The authors examined 51 B2 glenoids with mean retroversion of 19.1° treated with aTSA (all-polyethylene, pegged, cemented glenoid implant) and non-corrective, concentric reaming. At mean follow-up of 4.9 years, they noted that only two patients (3.9%) had glenoid component loosening, both of whom required revisions, but an overall implant survivorship rate of 95%. Similarly, Orvets et al.^41^ partially corrected B2 glenoid deformities with mean retroversion of 18° and 67% posterior subluxation using an eccentric reaming technique and noted excellent functional and radiographic outcomes at 50-months.^41^ Moreover, Hendel et al.^42^ previously reported that use of patient-specific instrumentation may result in greater accuracy (i.e., less over-reaming), more appropriate version correction and lower incidence of peg perforation when performing corrective reaming in the setting of a B2 glenoid.^42^ Our systematic review found that the use of an augmented glenoid component in the setting of posterior glenoid deficiency had the ability to obtain a mean retroversion correction from 21.8° to 9.5° post-operatively with low rates of radiographic and clinical complication at early- to mid-term follow-up.

Three studies^29,31,36^ included in our review reported the results of a full wedge augmented glenoid design (Equinoxe; Exactech, Gainesville, FL, USA). Wright et al. compared 24 age- and sex-matched patients treated with a full wedge posterior augmented glenoid to those treated with a standard glenoid component and eccentric reaming. They noted that 60% of posteriorly augmented shoulders demonstrated a radiolucent line with a mean radiographic glenoid line score of 1.1, whereas one-third of non-augmented shoulders were found to have a radiolucent line with mean radiographic glenoid line score of 0.438.^29^ However, no revision procedures were required and no significant differences in clinical outcomes were noted between the two groups at two-year follow-up. Priddy et al.^31^ found that greater degrees of augmentation (16° vs. 8° augment) accounted for half of all at-risk glenoids in their study with one of four shoulders requiring revision to hemi-arthroplasty for symptomatic aseptic loosening at three-year follow-up. The authors of this study discontinued the use of the 16° augment in favor of rTSA for cases of severe posterior glenoid deficiency.^31^ Grey et al.^36^ reported excellent clinical and radiographic results with an 8° full wedge glenoid augment (Equinoxe: Exactech, Gainesville, FL, USA) in 68 shoulders at 50-month follow-up with only two cases (2.9%) requiring revision for aseptic loosening of the glenoid component.^36^ While the 8° full wedge implant has shown promising short- to mid-term clinical and radiographic results, the 16° augment appears to have a higher rate of radiographic and clinical aseptic glenoid loosening. Overall, the use of a full wedge glenoid component (including both 8° and 16° augments) resulted in a low revision rate for aseptic loosening of 2.4% (3 out of 125) across the three eligible studies in this review.^29,31,36^

Four studies^30,32–34^ included in this review utilized a stepped glenoid component (Global StepTech, DePuy Synthes) with a weighted mean glenoid retroversion of 24.4°. Clinically, a statistically significant improvement was observed in both range of motion and patient reported outcomes across these studies. However, radiographically, there was a higher rate of lucency^32–34^ and peg perforation^30,33^ noted. Overall, the stepped glenoid design was found to have a 11.7% peg perforation rate. This translated to one aseptic loosening out of 112 shoulders (0.8%) demonstrating excellent short-term results (2.4 - to 3-year follow-up). However, the high rate of peg perforation does raise some concern for the long-term stability of the implant as peg perforation has been associated with poor clinical outcomes.^14^ The volume of bone removed during preparation of the glenoid for a stepped implant may contribute to this. Knowles et al. conducted a computational comparison of various augmented glenoid components including stepped, full wedge, and half wedge designs and noted the stepped design resulted in substantially greater volume of bone removal and poorer quality supporting bone.^43^

The current review demonstrates that the use of an augmented glenoid component (irrespective of design) to address severe posterior bone loss in patients undergoing aTSA is a promising solution to a challenging problem. However, these findings should be viewed with cautious optimism as they only represent early- to mid-term results. This is particularly important when viewed in the context of the excellent short- to mid-term outcomes reported with both corrective and non-corrective eccentric reaming.^40,41^ Furthermore, the degree of posterior glenoid deficiency included in this systematic review was moderate in severity with a mean retroversion of 21.8°. More severe bone loss and retroversion may preclude use of an aTSA. Likewise, use of an augmented glenoid component in milder deformity may result in excessive joint line lateralization and resultant overstuffing of the joint. As such, identifying the appropriate clinical setting and patient profile for use of an augmented glenoid component still requires further clarification. Lastly, this systematic review is comprised of levels III and IV studies, which makes it difficult to make any definitive conclusions or evidence-based recommendations from their findings. However, it is important to note that this review represents the best available clinical evidence on short-term outcomes with augmented glenoid use in aTSA.

## Conclusion

The use of an augmented glenoid component for the correction of posterior bone loss among patients undergoing aTSA was associated with low overall rates of complication (2.6%), revision surgery (1.9%) and excellent clinical outcomes at short-term follow-up. However, early reports demonstrated use of a 16° full-wedge glenoid component (Equinoxe; Exactech, Gainesville, FL, USA) may be related to a higher incidence of radiographic lucency and aseptic loosening, while the augmented 5-mm stepped design (Global StepTech, DePuy Synthes) may be associated with higher rates of peg perforation, raising some concern for long-term implant stability. As such, prospective studies examining mid- and long-term results as well as head-to-head comparisons between the various implant designs will help further elucidate safety and efficacy of these relatively new implants.

## References

[1] Day JS Lau E Ong KL , et al. Prevalence and projections of total shoulder and elbow arthroplasty in the United States to 2015. J Shoulder Elbow Surg 2010; 19: 1115–1120. 20554454 10.1016/j.jse.2010.02.009

[2] Kim SH Wise BL Zhang Y , et al. Increasing incidence of shoulder arthroplasty in the United States. J Bone Joint Surg Am 2011; 93: 2249–2254. 22258770 10.2106/JBJS.J.01994

[3] Wagner ER Farley KX Higgins I , et al. The incidence of shoulder arthroplasty: rise and future projections compared to hip and knee arthroplasty. J Shoulder Elbow Surg 2020; 29: 2601–2609. 33190759 10.1016/j.jse.2020.03.049

[4] Deshmukh AV Koris M Zurakowski D , et al. Total shoulder arthroplasty: long-term survivorship, functional outcome, and quality of life. J Shoulder Elbow Surg 2005; 14: 471–479. 16194737 10.1016/j.jse.2005.02.009

[5] Stephens SP Paisley KC Jeng J , et al. Shoulder arthroplasty in the presence of posterior glenoid bone loss. J Bone Joint Surg 2015; 97: 251–259. 25653326 10.2106/JBJS.N.00566

[6] Walch G Moraga C Young A , et al. Results of anatomic nonconstrained prosthesis in primary osteoarthritis with biconcave glenoid. J Shoulder Elbow Surg 2012; 21: 1526–1533. 22445158 10.1016/j.jse.2011.11.030

[7] Somerson JS Hsu JE Neradilek MB , et al. Analysis of 4063 complications of shoulder arthroplasty reported to the US Food and Drug Administration from 2012 to 2016. J Shoulder Elbow Surg 2018; 27: 1978–1986. 29759905 10.1016/j.jse.2018.03.025

[8] Walch G Badet R Boulahia A , et al. Morphologic study of the glenoid in primary glenohumeral osteoarthritis. J Arthroplasty 1999; 14: 756–760. 10512449 10.1016/s0883-5403(99)90232-2

[9] Michael J Bercik KK II . A modification to the Walch classification of the glenoid in primary glenohumeral osteoarthritis using three-dimensional imaging. J Shoulder Elbow Surg 2016; 25: 1601–1606. 27282738 10.1016/j.jse.2016.03.010

[10] Carroll RM Izquierdo R Vazquez M , et al. Conversion of painful hemiarthroplasty to total shoulder arthroplasty: long-term results. J Shoulder Elbow Surg 2004; 13: 599–603. 15570227 10.1016/j.jse.2004.03.016

[11] Getz CL Kearns KA Padegimas EM , et al. Survivorship of hemiarthroplasty with concentric glenoid reaming for glenohumeral arthritis in young, active patients with a biconcave glenoid. J Am Acad Orthop Surg 2017; 25: 715–723. 28953086 10.5435/JAAOS-D-16-00019

[12] Walch G Boulahia A Boileau P , et al. Primary glenohumeral osteoarthritis: clinical and radiographic classification. The Aequalis Group. *Acta Orthop Belg* 1998; 64: 46–52. 9922529

[13] Hsu JE Ricchetti ET Huffman GR , et al. Addressing glenoid bone deficiency and asymmetric posterior erosion in shoulder arthroplasty. J Shoulder Elbow Surg 2013; 22: 1298–1308. 23796384 10.1016/j.jse.2013.04.014

[14] Jason E Hsu SN . Glenoid Perforation With Pegged Components During Total Shoulder Arthroplasty. Orthopedics 2014; 37: 587–591. 10.3928/01477447-20140528-61 24972442

[15] Gates S Cutler H Khazzam M . Outcomes of posterior glenoid bone-grafting in anatomical total shoulder arthroplasty: a systematic review. JBJS Rev 2019; 7: e6–e6. 10.2106/JBJS.RVW.19.00005 31567619

[16] Neyton L Walch G Nov’e-Josserand L , et al. Glenoid corticocancellous bone grafting after glenoid component removal in the treatment of glenoid loosening. J Shoulder Elbow Surg 2006; 15: 173–179. 16517359 10.1016/j.jse.2005.07.010

[17] Iannotti JP Frangiamore SJ . Fate of large structural allograft for treatment of severe uncontained glenoid bone deficiency. J Shoulder Elbow Surg 2012; 21: 765–771. 22305919 10.1016/j.jse.2011.08.069

[18] Scalise J Iannotti JP . Bone grafting severe glenoid defects in revision shoulder arthroplasty. Clin Orthop Relat Res 2008; 466: 139–145. 18196386 10.1007/s11999-007-0065-7 PMC2505297

[19] Mizuno N Denard PJ Raiss P , et al. Reverse total shoulder arthroplasty for primary glenohumeral osteoarthritis in patients with a biconcave glenoid. J Bone Joint Surg Am 2013; 95: 1297–1304. 23864178 10.2106/JBJS.L.00820

[20] Favard L Berhouet J Colmar M , et al. Massive rotator cuff tears in patients younger than 65 years. What treatment options are available? *Orthop Traumatol Surg Res* 2009; 95: S19–S26. 19427282 10.1016/j.otsr.2009.03.005

[21] Guery J Favard L Sirveaux F , et al. Reverse total shoulder arthroplasty: survivorship analysis of eighty replacements followed for five to ten years. J Bone Joint Surg Am 2006; 88: 1742–1747. 16882896 10.2106/JBJS.E.00851

[22] Ek E Neukom L Catanzaro S , et al. Reverse total shoulder arthroplasty for massive irreparable rotator cuff tears in patients younger than 65 years old: results after five to fifteen years. J Shoulder Elbow Surg 2013; 22: 1199–1208. 23385083 10.1016/j.jse.2012.11.016

[23] Friedman LGM Garrigues GE . Anatomic augmented glenoid implants for the management of the B2 glenoid. J Shoulder Elbow Arthroplasty 2019; 3: 247154921987035–247154921987035. 10.1177/2471549219870350 PMC8282160 34497956

[24] Hermida JC Flores-Hernandez C Hoenecke HR , et al. Augmented wedge-shaped glenoid component for the correction of glenoid retroversion: a finite element analysis. J Shoulder Elbow Surg 2014; 23: 347–354. 24007648 10.1016/j.jse.2013.06.008

[25] Wang T Abrams GD Behn AW , et al. Posterior glenoid wear in total shoulder arthroplasty: eccentric anterior reaming is superior to posterior augment. Clin Orthop Relat Res 2015; 473: 3928–3936. 26242283 10.1007/s11999-015-4482-8 PMC4626525

[26] Sowa B Bochenek M Braun S , et al. Replacement options for the B2 glenoid in osteoarthritis of the shoulder: a biomechanical study. Arch Orthop Trauma Surg 2018; 138: 891–899. 29516162 10.1007/s00402-018-2915-z

[27] Ghoraishian M Abboud J Romeo A , et al. Augmented glenoid implants in anatomic total shoulder arthroplasty: review of available implants and current literature. J Shoulder Elbow Surg 2019; 28: 387–395. 30392937 10.1016/j.jse.2018.08.017

[28] Moher D Liberati A Tetzlaff J , et al. Preferred reporting items for systematic reviews and meta-analyses: the PRISMA statement. BMJ 2009; 339: b2535–b2535. 19622551 10.1136/bmj.b2535 PMC2714657

[29] Thomas W Wright SGG . Preliminary results of a posterior augmented glenoid compared to an all polyethylene standard glenoid in anatomic total shoulder arthropalsty. Bull Hosp Jt Dis 2015; 73: 79–85. 26631201

[30] Ko JW Syed UA Barlow JD , et al. Comparison of asymmetric reaming versus a posteriorly augmented component for posterior glenoid wear and retroversion: a radiographic study. Arch Bone Jt Surg 2019; 7: 307–313. 31448306 PMC6686063

[31] Priddy M Zarezadeh A Farmer KW , et al. Early results of augmented anatomic glenoid components. J Shoulder Elbow Surg 2019; 28: S138–S145. 31196508 10.1016/j.jse.2019.04.014

[32] Favorito PJ Freed RJ Passanise AM , et al. Total shoulder arthroplasty for glenohumeral arthritis associated with posterior glenoid bone loss: results of an all-polyethylene, posteriorly augmented glenoid component. J Shoulder Elbow Surg 2016; 25: 1681–1689. 27212072 10.1016/j.jse.2016.02.020

[33] Stephens SP Spencer EE Wirth MA . Radiographic results of augmented all-polyethylene glenoids in the presence of posterior glenoid bone loss during total shoulder arthroplasty. J Shoulder Elbow Surg 2017; 26: 798–803. 27887871 10.1016/j.jse.2016.09.053

[34] Ho JC Amini MH Entezari V , et al. Clinical and radiographic outcomes of a posteriorly augmented glenoid component in anatomic total shoulder arthroplasty for primary osteoarthritis with posterior glenoid bone loss. J Bone Joint Surg Am 2018; 100: 1934–1948. 30480598 10.2106/JBJS.17.01282

[35] Das AK Wright AC Singh J , et al. Does posterior half-wedge augmented glenoid restore version and alignment in total shoulder arthroplasty for the B2 glenoid? J Clin Orthop Trauma 2020; 11: S275–S279. 32189953 10.1016/j.jcot.2020.02.005 PMC7067996

[36] Grey SG Wright TW Flurin PH , et al. Clinical and radiographic outcomes with a posteriorly augmented glenoid for Walch B glenoids in anatomic total shoulder arthroplasty. J Shoulder Elbow Surg 2020; 29: e185–e195. 31924515 10.1016/j.jse.2019.10.008

[37] Terrier A Goetti P Becce F , et al. Reduction of scapulohumeral subluxation with posterior augmented glenoid implants in anatomic total shoulder arthroplasty: short-term 3D comparison between pre- and post-operative CT. Orthop Traumatol Surg Res 2020; 106: 681–686. 32284278 10.1016/j.otsr.2020.03.007

[38] Lazarus M Jensen K Southworth C , et al. The radiographic evaluation of keeled and pegged glenoid component insertion. J Bone Joint Surg Am 2002; 84: 1174–1182. 12107318 10.2106/00004623-200207000-00013

[39] Clavert P Millett PJ Warner JJ . Glenoid resurfacing: what are the limits to asymmetric reaming for posterior erosion? J Shoulder Elbow Surg 2007; 16: 843–848. 18061118 10.1016/j.jse.2007.03.015

[40] Grantham WJ Dekker TJ Lacheta L , et al. Total shoulder arthroplasty outcomes after noncorrective, concentric reaming of B2 glenoids. JSES Int 2020; 4: 644–648. 32939500 10.1016/j.jseint.2020.04.006 PMC7479043

[41] Orvets ND Chamberlain AM Patterson BM , et al. Total shoulder arthroplasty in patients with a B2 glenoid addressed with corrective reaming. J Shoulder Elbow Surg 2018; 27: S58–S64. 29501223 10.1016/j.jse.2018.01.003

[42] Hendel MD Bryan JA Barsoum WK , et al. Comparison of patient-specific instruments with standard surgical instruments in determining glenoid component position: a randomized prospective clinical trial. J Bone Joint Surg 2012; 94: 2167–2175. 23224387 10.2106/JBJS.K.01209

[43] Knowles NK Ferreira LM Athwal GS . Augmented glenoid component designs for type B2 erosions: a computational comparison by volume of bone removal and quality of remaining bone. J Shoulder Elbow Surg 2015; 24: 1218–1226. 25648971 10.1016/j.jse.2014.12.018

